# Escherichia coli ST131-*H*22 as a Foodborne Uropathogen

**DOI:** 10.1128/mBio.00470-18

**Published:** 2018-08-28

**Authors:** Cindy M. Liu, Marc Stegger, Maliha Aziz, Timothy J. Johnson, Kara Waits, Lora Nordstrom, Lori Gauld, Brett Weaver, Diana Rolland, Sally Statham, Joseph Horwinski, Sanjeev Sariya, Gregg S. Davis, Evgeni Sokurenko, Paul Keim, James R. Johnson, Lance B. Price

**Affiliations:** a Antibiotic Resistance Action Center, Department of Environmental and Occupational Health, Milken Institute School of Public Health, George Washington University, Washington, DC, USA; b Center for Microbial Genetics and Genomics, Northern Arizona University, Flagstaff, Arizona, USA; c Division of Pathogen Genomics, Translational Genomics Research Institute, Flagstaff, Arizona, USA; d Bacteria, Parasites and Fungi, Statens Serum Institut, Copenhagen, Denmark; e Department of Veterinary and Biomedical Sciences, College of Veterinary Medicine, University of Minnesota, St. Paul, Minnesota, USA; f Flagstaff Medical Center, Flagstaff, Arizona, USA; g Department of Microbiology, University of Washington School of Medicine, Seattle, Washington, USA; h Minneapolis Veterans Affairs Health Care System, Minneapolis, Minnesota, USA; i University of Minnesota, Minneapolis, Minnesota, USA; University of Michigan Medical School; University of Maryland School of Medicine

**Keywords:** Antibiotic resistance, antimicrobial resistance, ColV plasmid, *Escherichia coli*, ExPEC, foodborne, host adaptation, poultry, ST131, UTI, urinary tract infection

## Abstract

Escherichia coli sequence type 131 (ST131) has emerged rapidly to become the most prevalent extraintestinal pathogenic E. coli clones in circulation today. Previous investigations appeared to exonerate retail meat as a source of human exposure to ST131; however, these studies focused mainly on extensively multidrug-resistant ST131 strains, which typically carry allele 30 of the *fimH* type 1 fimbrial adhesin gene (ST131-*H*30). To estimate the frequency of extraintestinal human infections arising from foodborne ST131 strains without bias toward particular sublineages or phenotypes, we conducted a 1-year prospective study of E. coli from meat products and clinical cultures in Flagstaff, Arizona. We characterized all isolates by multilocus sequence typing, *fimH* typing, and core genome phylogenetic analyses, and we screened isolates for avian-associated ColV plasmids as an indication of poultry adaptation. E. coli was isolated from 79.8% of the 2,452 meat samples and 72.4% of the 1,735 culture-positive clinical samples. Twenty-seven meat isolates were ST131 and belonged almost exclusively (*n* = 25) to the ST131-*H*22 lineage. All but 1 of the 25 H22 meat isolates were from poultry products, and all but 2 carried poultry-associated ColV plasmids. Of the 1,188 contemporaneous human clinical E. coli isolates, 24 were ST131-*H*22, one-quarter of which occurred in the same high-resolution phylogenetic clades as the ST131-*H*22 meat isolates and carried ColV plasmids. Molecular clock analysis of an international ST131-*H*22 genome collection suggested that ColV plasmids have been acquired at least six times since the 1940s and that poultry-to-human transmission is not limited to the United States.

## Abstract (precis)

E. coli ST131 is an important extraintestinal pathogen that can colonize the gastrointestinal tracts of humans and food animals. Here, we combined detection of accessory traits associated with avian adaptation (ColV plasmids) with high-resolution phylogenetics to quantify the portion of human infections caused by ST131 strains of food animal origin. Our results suggest that one ST131 sublineage—ST131-*H*22—has become established in poultry populations around the world and that meat may serve as a vehicle for human exposure and infection. ST131-*H*22 is just one of many E. coli lineages that may be transmitted from food animals to humans. Additional studies that combine detection of host-associated accessory elements with phylogenetics may allow us to quantify the total fraction of human extraintestinal infections attributable to food animal E. coli strains.

## INTRODUCTION

Escherichia coli causes millions of extraintestinal infections in the United States each year, including urinary tract infections (UTIs) (1, 2). In contrast to bladder infections, which typically amount to little more than a painful annoyance, invasive UTIs that involve the kidneys and bloodstream can be life-threatening (3). The emergence of extensively antimicrobial-resistant E. coli strains has increased the hazard posed by these common infections (4, 5). The potential for food animal E. coli strains to make their way to humans via contaminated meat and to cause UTIs was proposed as early as 1970 (6). Despite early supportive studies (7–11), the concept did not gain popularity until more than two decades later, when separate UTI outbreaks in Denmark, Canada, and the United States were postulated to have been caused by foodborne E. coli strains (12–15). In subsequent years, many studies have shown that extraintestinal pathogenic E. coli strains routinely colonize food animals and contaminate the food supply (16–19). The likely link between foodborne E. coli and human urinary tract infections underscores the public health relevance of use of antibiotics in food animal production, which was recognized by researchers from the very beginning (6, 20).

Antibiotic use in food animal production, which is well established as contributing to antibiotic resistance in classical foodborne pathogens such as *Salmonella* and *Campylobacter* (21, 22), more recently has been linked to the emergence of antibiotic-resistant colonizing opportunistic pathogens (COPs) such as Staphylococcus aureus and Clostridium difficile in food animals and humans (23). Since its discovery in the early 2000s, methicillin-resistant S. aureus (MRSA) sequence type 398 (ST398) has become a model for how antibiotic use in food animal production can fuel the emergence of novel COP strains that can spread to humans through occupational exposure and retail meat (24–27). Likewise, compelling evidence now links multiple, antimicrobial-resistant substrains of C. difficile ribotype 078 (RT078) to antimicrobial use in food animal production (28). However, of all the COPs, those from *Enterobacteriaceae* may threaten human health most, due to their broad host range and frequent exchange of mobile resistance elements. This potential risk was highlighted by the discovery of the colistin resistance gene *mcr-1* in multiple E. coli strains from food animals, retail meat, and people (29).

There is a need to quantify the human disease burden associated with E. coli from food animals. A combination of phylogenetic analysis based on single nucleotide polymorphisms (SNPs) from the E. coli core genome plus information regarding host-adaptive accessory elements could enable investigators to more accurately identify the host origins of isolates and infer directionality of such transmissions.

ColV plasmids have been recognized as a defining trait of avian pathogenic E. coli (APEC) (30). These plasmids are self-transmissible F-type plasmids that harbor numerous traits demonstrated to enhance the virulence and fitness of APEC strains in colonizing and infecting commercial broiler chickens and turkeys (31). Collectively, ColV plasmids and their virulence genes have been identified in greater than 80% of E. coli isolated from diseased poultry, and they are highly predictive of the APEC pathotype (32). In contrast, ColV plasmids are rarely found in human clinical E. coli isolates, and we hypothesize that their presence in such isolates indicates evidence of historical zoonotic transmission from poultry to humans (33).

E. coli ST131 has emerged explosively since the early 2000s to become the most important multidrug-resistant uropathogen in circulation today (5, 34). High-resolution phylogenetic analyses have identified multiple lineages within ST131 (35, 36), each associated with a specific allele of the type 1 fimbrial adhesin gene (*fimH*), including the *H*22 and *H*27, *H*30, and *H*41 lineages. While ST131-*H*30 is the most prevalent and extensively resistant of these ST131 lineages, all four are important etiologic agents for community-acquired UTI (37–39). The ST131-*H*22 lineage is of particular interest because of its ability to colonize poultry, contaminate meat, and carry the *mcr-1* and *mcr-3* mobile resistance determinants that confer resistance to colistin, the only available active agent against certain extensively resistant Gram-negative pathogens (29, 40, 41).

Although previous studies suggested that food is not an important reservoir of E. coli ST131 (19, 42), these studies focused on the better-known ST131-*H*30 lineage, and so those findings may not accurately represent the epidemiology of other ST131 lineages, including ST131-*H*22. Therefore, to evaluate food as a potential source of UTI caused by E. coli ST131-*H*22, we collected E. coli isolates from human extraintestinal infections and retail meat products in a small, geographically isolated American city over the course of 1 year and then compared the two E. coli populations by using a unique combination of high-resolution phylogenetic analyses and ColV plasmid interrogation.

## RESULTS

### Prevalence of E. coli ST131 among meat and human clinical isolates.

During the 12-month study period, E. coli was recovered from 72.4% of the 1,735 positive clinical urine and blood cultures and from 81.7% of the 2,460 meat products. In total, 1,923 meat isolates and 1,188 human clinical isolates were sequenced successfully and were assigned to 443 E. coli STs. Of the 443 STs, 247 STs included only meat isolates (785 [40.8%] of meat isolates), 120 STs included only clinical isolates (403 [33.9%] of human clinical isolates), and 76 STs included both meat and clinical isolates (1,138 [59.2%] of meat isolates and 785 [66.1%] of clinical isolates). ST131 was detected among both meat and human clinical isolates, and it was the single most common ST among the clinical E. coli isolates (182/1,188 [15.3%]) and 13th among meat isolates (25/1,923 [1.3%]).

### Phylogenetic relatedness of E. coli ST131 isolates from meat and human clinical specimens.

**FIG 1:**
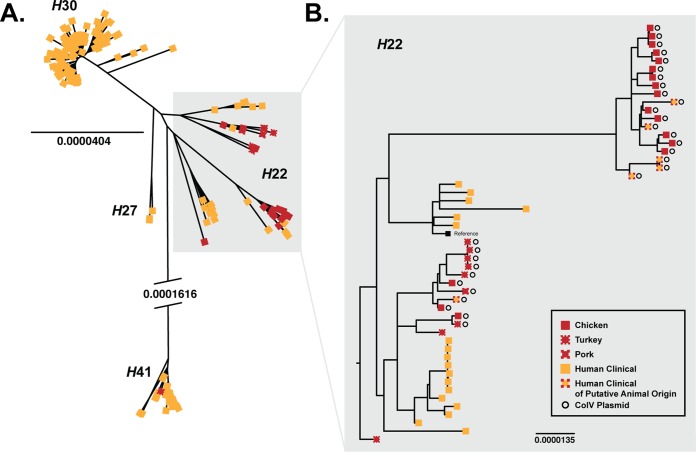
Whole-genome phylogeny of meat and human clinical Escherichia coli ST131 isolates. (A) A total of 207 ST131 isolates from meat (in red) and human clinical specimens (in yellow) were included in this unrooted phylogeny. The *fimH* alleles corresponded closely with the phylogenetic groupings and were used to designate the four major lineages: *H*22, *H*27, *H*30, and *H*41. The ST131-*H*22 lineage included isolates with other *fimH* alleles *fimH161* or *fimH207*, both of which are minor sequence variants of *fimH22*. Most (69%) of the clinical isolates belonged to the ST131-*H*30 lineage, with the balance falling into the ST131-*H*41 (16%), ST131-*H*22 (13%), and ST131-*H*27 (2%) lineages. In contrast, 93% of the meat isolates belonged to the ST131-*H*22 lineage. (B) Forty-nine ST131-*H*22 isolates from meat (in red) and human clinical specimens (in yellow) were included in this rooted high-resolution phylogeny. While some subclades contained only human clinical isolates, two subclades included intermingled meat and human clinical isolates. All ColV plasmid-positive ST131-*H*22 human clinical isolates fell within the latter two subclades, suggesting that these meat and human clinical isolates were derived from a common source. The scale bar represents the substitution rate in the conserved core genome. The branch leading to the *H*41 clade is truncated to approximately 20% of its full length for graphic purposes.

The genetic relationships between E. coli ST131 isolates from meat and human clinical specimens were examined using a core genome SNP-based phylogenetic analysis. This total ST131 population exhibited four major lineages that corresponded to the four major *fimH* alleles of ST131, i.e., *fimH*22, *fimH*27, *fimH*30, and *fimH*41. One of these lineages, ST131-*H*22, included nearly all of the ST131 meat isolates (24/25 [96%]), plus 24 (13%) of the ST131 human clinical isolates (Fig. 1A).

### Patient demographics and clinical presentation. 

Patient demographics varied significantly by ST131 lineage. Specifically, compared to patients with an ST131-*H*30 clinical isolate (*n* = 125), patients with an ST131-*H*22 clinical isolate (*n* = 24) had a significantly lower median age (47 years, with interquartile range [IQR] of 37, versus 65 years and an IQR of 33: *P* = 0.008) and a higher female proportion (87.5% versus 76.0%; *P* = 0.29).

In contrast, among the 88 ST131 isolates (48% of 182) for which clinical data were available, clinical presentation did not vary significantly by ST131 lineage. The overall syndrome distribution was 48% asymptomatic bacteriuria, 32% cystitis, 17% pyelonephritis, and 5% urosepsis. This distribution did not differ significantly between the 16 ST131-*H*22 isolates (67% of 24) and 52 ST131-*H*30 isolates (42% of 125) for which clinical data were available, i.e., 38% versus 56% asymptomatic bacteriuria, 44% versus 25% cystitis, 19% versus 14% pyelonephritis, and 0% versus 6% urosepsis, respectively.

### Avian-associated ColV plasmids and phylogenetic analysis of *H*22 human clinical isolates.

High-resolution phylogenetic analysis of the ST131-*H*22 lineage revealed multiple distinct sublineages, two of which showed particularly close phylogenetic similarity between human clinical and meat isolates (Fig. 1B). Ninety-three percent of meat source ST131-*H*22 isolates were from poultry products; therefore, a potential link between poultry and human clinical isolates was explored further based on the presence of ColV plasmids, because ColV plasmids have been associated with E. coli fitness and virulence in avian hosts and are a defining trait of avian pathogenic E. coli (43). Plasmid profiling and draft genome comparisons identified a presumptive ColV plasmid in 84% of meat source and 25% of human clinical source ST131-*H*22 isolates (see Fig. S2 in the supplemental material). In the core genome phylogeny, human clinical isolates carrying ColV plasmids were more closely related to meat isolates carrying ColV plasmids than they were to human clinical isolates without the plasmids, further supporting poultry as a source for the ST131-*H*22 human clinical isolates (Fig. S2).

10.1128/mBio.00470-18.1 FIG S1  Diagnosis assignment algorithm used to classify the clinical diagnosis associated with each E. coli-positive human clinical culture as asymptomatic bacteriuria, cystitis, pyelonephritis, or urosepsis. Download FIG S1, PDF file, 0.2 MB. Copyright © 2018 Liu et al. 2018 Liu et al. This content is distributed under the terms of the Creative Commons Attribution 4.0 International license.

10.1128/mBio.00470-18.2 FIG S2  ColV plasmid gene content among Escherichia coli ST131-*H*22 isolates from human clinical specimens and meat. ST131-*H*22 isolates from both human clinical specimens (yellow box) and meat (red box) shared substantial ColV plasmid gene content (black box). The meat and human clinical ST131-*H*22 isolates were intermingled extensively according to hierarchal clustering based on plasmid gene content, demonstrating their considerable genetic similarity with respect to signature ColV-plasmid elements. An isolate was considered to be ColV plasmid positive if it had at least one gene from four or more of the following gene clusters: (i) *cvaABC* and *cvi* (the ColV operon), (ii) *iroBCDEN* (the salmochelin operon), (iii) *iucABCD* and *iutA* (the aerobactin operon), (iv) *etsABC*, (v) *ompT* and *hlyF*, and (vi) *sitABCD*. ColV plasmids (black open circles) were identified in 25% of the human clinical ST131-*H*22 isolates and in 84% of meat ST131-*H*22 isolates from Flagstaff. Download FIG S2, PDF file, 0.3 MB. Copyright © 2018 Liu et al. 2018 Liu et al. This content is distributed under the terms of the Creative Commons Attribution 4.0 International license.

### ColV plasmids and antimicrobial susceptibility in *H*22 isolates.

ColV plasmid-positive ST131-*H*22 isolates were more likely to be multidrug resistant than were ColV plasmid-negative ST131-*H*22 isolates (Table 1). Furthermore, ColV plasmid-carrying isolates were significantly more likely than ColV plasmid-negative isolates to be resistant to tetracycline and gentamicin, two antibiotics commonly used in U.S. food animal production (44). Consistent with resistance phenotypes, resistance-conferring genes were also more prevalent among ColV plasmid-positive isolates than among ColV plasmid-negative isolates (Data Set S2).

**TABLE 1:** Antimicrobial resistance profiles of 49 ST131-*H*22 Escherichia coli isolates from retail meats and human clinical specimens in relation to isolate source and ColV plasmid status

Antimicrobial	No. (%) resistant in:				Logistic regression [OR (95% CI)]		
	Meat		Clinical specimen		Univariate		Multivariate, isolate source and ColV status
	ColV^+^ (*n* = 21)	ColV^−^ (*n* = 4)	ColV^+^ (*n* = 6)	ColV^−^ (*n* = 18)	Meat source	ColV^+^	
Ampicillin	17 (81)	3 (75)	2 (33)	7 (39)	6.7 (2.0, 26.1)	2.9 (0.9, 9.6)	NA^a^
Ampicillin-sulbactam	13 (62)	3 (75)	2 (33)	6 (33)	3.6 (1.1, 12.1)	1.8 (0.6, 5.8)	NA
Cefazolin	17 (81)	3 (75)	1 (17)	4 (22)	15.2 (4.1, 68.2)	4.3 (1.3, 15.0)	Meat: 15.6 (3.2, 118.3); ColV^+^: 1.0 (0.1, 4.9)
Cefoxitin	4 (19)	0 (0)	1 (17)	2 (11)	1.3 (0.3, 7.5)	2.3 (0.4, 17.1)	NA
Ceftriaxone	4 (19)	1 (25)	1 (17)	0 (0)	5.7 (0.8, 115.1)	4.8 (0.7, 95.5)	NA
Ciprofloxacin	0 (0)	0 (0)	0 (0)	1 (6)	NA	NA	NA
Gentamicin	8 (38)	0 (0)	1 (17)	1 (6)	5.2 (1.1, 37.4)	10.5 (1.7, 203.3)	Meat source: 2.1 (0.3, 17.9); ColV^+^: 7.0 (0.8, 153.7)
Trimethoprim-sulfamethoxazole	0 (0)	0 (0)	1 (17)	0 (0)	NA	NA	NA
Tetracycline	11 (52)	3 (75)	3 (50)	0 (0)	8.9 (2.3, 45.0)	6.8 (1.8, 34.1)	Meat source: 5.2 (1.1, 30.9); ColV^+^: 2.9 (0.6, 17.3)
≥2 antimicrobial classes^b^	19 (91)	3 (75)	3 (50)	7 (39)	10.3 (2.7, 52.4)	5.3 (1.5, 20.6)	Meat source: 7.1 (1.5, 44.2); ColV^+^: 2.0 (0.4, 9.4)
≥3 antimicrobial classes^c^	17 (81)	3 (75)	2 (33)	3 (17)	15.2 (4.1, 68.2)	6.3 (1.9, 23.7)	Meat source: 10.7 (2.4, 60.4); ColV^+^: 2.0 (0.4, 9.4)

a NA, not applicable.

b Resistance to 2 or more antimicrobial classes.

c Resistance to 3 or more antimicrobial classes.

### Natural history of ColV plasmids in ST131-*H*22.

**FIG 2:**
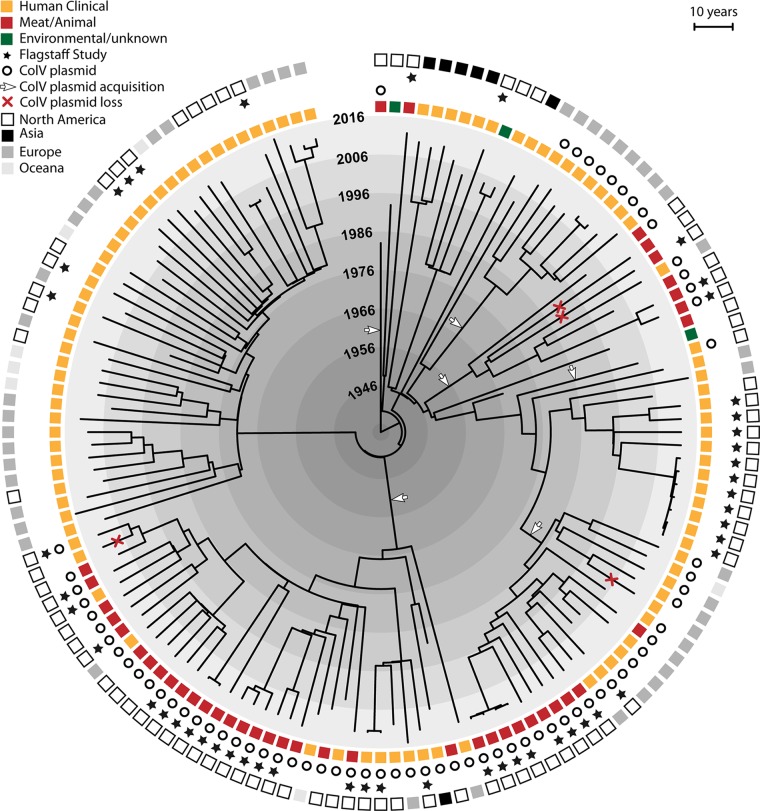
Bayesian analysis of 140 global Escherichia coli ST131-*H22* genomes. The tree, based on a total of 3,774 SNPs identified in the relaxed core genome of 49 Flagstaff ST131-*H*22 isolates and 91 international H22 isolates, was obtained using a strict clock model, GTR substitution rate, and the Bayesian Skyline population tree model. The analysis strongly indicates six independent acquisitions of ColV plasmids within the ST131-*H*22 sublineage. The tips of the tree are constrained by year of isolation, with shaded areas used to represent the time scale (in decades). White arrows and red crosses point to branches associated with putative ColV plasmid acquisitions and losses, respectively.

As noted above, multiple ST131-*H*22 sublineages carry ColV plasmids. Three independent statistical modeling approaches—including coalescence-based analysis using a strict molecular clock, the GTR substitution model, and the Bayesian Skyline tree prior—suggested that ColV plasmids were acquired by members of the *H*22 clade in at least six separate genetic events since the mid-1940s (Fig. 2). Three of the six ColV plasmid-positive lineages showed intermingling of human and meat isolates. In contrast, another of these lineages consisted solely of human isolates. This clade’s eight human isolates were all from Europe, where meat products were not sampled in parallel with humans, likely decreasing the opportunity for finding genomic commonality between meat and human isolates.

Analysis of the international collection of ST131-*H*22 genomes identified 22 additional ColV plasmid-carrying human isolates, indicating that putative poultry-to-human transmission is not limited to Flagstaff, Arizona, but likely is a global phenomenon.

### Mobile colistin resistance in ST131-*H*22. 

Two published ST131-*H*22 isolates were identified previously as carrying mobile colistin resistance (*mcr*) determinants (40, 41). Our genomic analysis showed that both carry ColV plasmids. When they were added to the ST131-*H*22 core genome phylogram, each was assigned to an ST131-*H*22 clade that included intermingled human and meat isolates (Fig. S3).

10.1128/mBio.00470-18.3 FIG S3  Maximum likelihood phylogeny of global ST131-*H*22 isolates, showing placement of *mcr*-positive strains among those found in Enterobase, Flagstaff isolates, and two *mcr*-positive isolates from the Danish national surveillance collection at Statens Serum Institut. Both *mcr*-positive isolates were ColV positive and clustered in ColV-positive clades. The scale bar represents the substitution rate in the conserved core genome. Download FIG S3, PDF file, 0.3 MB. Copyright © 2018 Liu et al. 2018 Liu et al. This content is distributed under the terms of the Creative Commons Attribution 4.0 International license.

## DISCUSSION

Our analysis of concurrently collected meat and clinical isolates from Flagstaff showed that ST131-*H*22 accounted for nearly all of the ST131 meat isolates and for 13% of the ST131 clinical isolates, mostly from patients with cystitis and pyelonephritis. Importantly, whole-genome-based phylogenetic analysis showed that several subsets of ST131-*H*22 clinical isolates were closely related to meat isolates and carried avian-associated ColV plasmids (45, 46). Taken together, these findings support that a nonnegligible subset of community-acquired E. coli ST131 UTI episodes in the United States is foodborne.

Coalescence analysis suggests that the ST131-*H*22 lineage has acquired ColV plasmids at least six times, dating back as far as the 1940s. We assumed, for maximal parsimony, that when most isolates within a phylogenetic clade were ColV positive, a common ancestor to that clade acquired a ColV plasmid in a single genetic event and that any ColV-negative isolates within the clade resulted from plasmid loss events. The consistency of ColV plasmid carriage within some of the corresponding sublineages suggests that such plasmids have remained under selection—possibly in poultry flocks—for decades. If ST131-*H*22 strains have been circulating in poultry flocks since the 1940s, countless spillover events from poultry to humans could have occurred over the past several decades.

Three of the ColV plasmid-positive sublineages showed intermingling among isolates of human and poultry origin (Fig. 2). One exception was a sublineage composed exclusively of European human isolates. This clade’s polarized source distribution may reflect a bias in EnteroBase toward European human isolates and away from poultry isolates, rather than the establishment of a ColV plasmid-positive ST131-*H*22 sublineage in humans, although this possibility cannot be excluded. Further sampling of poultry flocks or meat from Europe is needed to investigate this question further.

Our findings support the hypothesis that the ST131-*H*22 strains that contaminate retail poultry products originate from food animal populations rather than humans. If human source contamination were a significant contributor to ST131 on retail meat products, one would anticipate a more equal distribution of ST131 lineages, including ST131-*H*30. Alternatively, compared to other ST131 sublineages, ST131-*H*22 strains may have better survival in the processing plant environment or on poultry products, but this seems less likely.

The evolving story of mobile colistin resistance elements among livestock-associated E. coli strains underscores the importance of our findings. Shortly after the discovery of *mcr-1* in isolates from Chinese livestock and people (29), multiple short reports described the retrospective detection of this element and other variants (*mcr-2* and -*3*) from countries around the world (47–53). The first such report was from Denmark, where investigators identified an *mcr-1*-positive extended-spectrum β-lactamase (ESBL)-producing E. coli strain that had been isolated in 2012 from an imported poultry product (40). Subsequently, this isolate was discovered to represent the ST131-*H*22 lineage. Likewise, in 2017, an ESBL-producing Danish bloodstream isolate from 2014 was reported that represened the ST131-*H*22 lineage and carry *mcr-3* (41). The striking diversity of E. coli strains found to carry the *mcr* genes provides empirical evidence of the associated mobile elements’ promiscuity. The fact that the gene has found its way into ST131-*H*22—a lineage capable of jumping hosts and causing serious extraintestinal infections in humans—may make it difficult to prevent the further spread of this element from livestock to humans.

Our study had several notable strengths that enabled us to estimate the relatedness of ST131 strains from meat products and clinical specimens. First, we isolated E. coli samples without bias toward any particular genotype or phenotype. Second, we collected concurrent clinical isolates and retail meat samples in the same geographically isolated community. Third, we systematically sampled all available brands of poultry and pork sold in the city, twice monthly, for a full year. Fourth, we analyzed all publicly available ST131 genomes from meat, animal, and clinical isolates via a high-resolution, whole-genome sequence-based approach. Previous studies of foodborne UTI caused by ST131 focused on ST131-*H*30 (54, 55) or relied on convenience-based sampling (19, 56, 57), which limited their ability to assess foodborne transmissions of non-*H*30 ST131 strains. Such studies also were limited by their use of strain typing methods (58) that did not provide the same phylogenetic resolution as does whole-genome sequencing (35).

Our study also had limitations. First, we did not collect data on the shopping and consumption habits of the source patients for the clinical isolates; this precluded a search for correlations between the strains recovered from particular meat brands versus from the people who purchased those brands, although such correlations likely could be obscured by person-to-person transmission of strains initially from a food source (59). Second, our analyses of ST131-*H*22 isolates outside Flagstaff were based on publicly available genome sequences that were not systematically collected, which precluded their use to assess host origins. However, these isolates did provide the temporal diversity necessary to calibrate our molecular clock analyses. Finally, while we have provided strong evidence that a substantial portion of the ST131-*H*22 strains infecting humans originate from poultry, our study was insufficient to determine definitively whether individual infections arose from direct exposure to contaminated poultry or from human-to-human transmission following the exposure to contaminated poultry. Despite these limitations, this study provides important new insights into the epidemiology of extraintestinal E. coli infections.

In summary, our findings demonstrate the potential for E. coli ST131-*H*22 to serve as a foodborne uropathogen. ST131 is just one of many STs that are potentially transmitted from food animals to humans. Therefore, while poultry ST131-*H*22 may represent only a small fraction of human infections, the fraction of human extraintestinal disease due to food animal E. coli may be much higher.

## MATERIALS AND METHODS

### Study design.

We conducted a 12-month study from January to December 2012 to determine the degree of phylogenomic overlap between contemporaneous E. coli ST131 isolates from retail meat products and human clinical specimens in Flagstaff, Arizona. All available brands of chicken, turkey, and pork were sampled every 2 weeks from the nine major grocery chains in Flagstaff. (For chains with multiple outlets, only one store per chain was sampled.) During the same 12-month period, bacterial isolates from all positive urine and blood cultures were collected from Flagstaff Medical Center (the main clinical laboratory servicing northern Arizona). For positive urine and blood cultures from patients residing within Flagstaff (est. population of 65,870, according to the 2010 U.S. Census), medical records were reviewed to determine whether the patient had asymptomatic bacteriuria, cystitis, pyelonephritis, or urosepsis, according to the algorithm in Fig. S1. The Northern Arizona Healthcare IRB approved isolate collection and medical record review (protocol number 573857-4) with a waiver of consent.

### E. coli recovery from retail meats and human urine and blood cultures.

E. coli was recovered from meat products in the research laboratory using enrichment methods as described previously (60). Up to four putative E. coli isolates per sample were identified and purified on HardyCHROM UTI agar (Hardy Diagnostics, Santa Maria, CA, USA). One E. coli isolate per positive sample was selected at random for subsequent analysis.

E. coli urine and blood isolates were recovered in the clinical laboratory using standard techniques. Briefly, urine specimens were collected by midstream clean catch or straight catheterization and cultured on sheep blood and MacConkey agars within 2 h of collection or with refrigeration for up to 24 h. A positive urine culture was defined as ≥10^4^ CFU per ml of urine for clean catch or ≥10^3^ CFU/ml for straight catheterization specimens. Species determination was done using the BD Phoenix instrument (Becton, Dickinson Diagnostic Systems, Sparks, MD, USA).

### Antimicrobial susceptibility testing.

The susceptibility of meat isolates to ampicillin, ampicillin-sulbactam, cefazolin, cefoxitin, ceftriaxone, ciprofloxacin, gentamicin, nitrofurantoin, trimethoprim-sulfamethoxazole, and tetracycline was determined by disk diffusion in accordance with procedures and breakpoints recommended by the Clinical and Laboratory Standards Institute (61). The susceptibility of clinical isolates to the same agents was determined using the BD Phoenix instrument per the manufacturer’s instructions. In the subsequent analysis, intermediate isolates were classified as resistant, and isolates resistant to ≥3 drug classes were classified as multidrug resistant.

### Multilocus sequence typing.

As part of a larger study on foodborne *Enterobacteriaceae*, isolates were subjected to whole-genome sequencing on the Illumina HiSeq system (Illumina, San Diego, CA) (60). All Illumina DNA sequence libraries used in this study are available on the NCBI Sequence Read Archive (accession number PRJNA407956). Resultant sequences were analyzed for multilocus sequence typing (MLST) housekeeping genes by BLAST (v.2.2.25+) (62) at 100% query coverage and nucleotide identity. Results were matched against an MLST reference database to identify ST131 isolates (63).

### *fimH* typing.

Allelic variants of *fimH* were identified by assembling the Illumina short-read sequences into contigs by using SPAdes (v.3.5) (64). After quality checks using QUAST (v.2.3) (65), each assembled genome was compared to an in-house *fimH* allele reference database using BLASTN (v.2.2.25+) (66). *fimH* allele assignments were made based on 100% query coverage and nucleotide identity.

### Whole-genome-based phylogenetic analysis.

To determine the genetic relatedness of E. coli ST131 human clinical and meat isolates, SNPs from the core genome were used to construct a maximum likelihood phylogeny for all ST131 isolates. Briefly, using the NASP pipeline (v.1.0.0) (67), Illumina short-read sequences were aligned to a published ST131 chromosome (strain JJ1887; GenBank accession number CP014316) (68) by using BWA-MEM (v.0.7.12) (69), and SNPs were called using GATK (v.3.5) (70). After removing recombinant regions using Gubbins (v.2.1) (71), the resultant SNP matrices were used to construct phylogenetic trees in PhyML with Smart Model selection (v.3.0) (72), and support values were calculated by bootstrap sampling (*n* = 100). Reads from ST131-*H*22 isolates were aligned to a published ST131-*H*22 chromosome (strain SaT040; GenBank accession number CP014495) (73) and were analyzed as described above.

### ColV plasmid profiling.

Partially assembled FUTI genomes were compared to a published avian ColV plasmid (A2363 pAPEC-O2-ColV; GenBank accession number NC_007675) using nucleotide-nucleotide BLAST (v.2.2.25+) (66). Hits were determined with a ≥ 90% cutoff for similarity and ≥95% length coverage with a maximum gap size of 5 and a maximum of 2 gaps. An isolate was considered ColV plasmid positive if it had at least one gene from four or more of the following sets: (i) *cvaABC* and *cvi* (the ColV operon), (ii) *iroBCDEN* (the salmochelin operon), (iii) *iucABCD* and *iutA* (the aerobactin operon), (iv) *etsABC*, (v) *ompT* and *hlyF*, and (vi) *sitABCD*.

### Coalescence-based analyses.

To investigate the emergence of ColV plasmids across the ST131-*H*22 lineage, a temporal analysis was performed on the ST131-*H*22 isolates from Flagstaff (*n* = 49) and a global collection of all available ST131-*H*22 genome sequences, as identified on 1 April 2017 at the EnteroBase repository (*n* = 91) (http://enterobase.warwick.ac.uk), which included information on collection year, source, and location (Data Set S1). To reduce distortion of the analysis by recombination, an SNP matrix was generated against ST131-*H*22 reference strain SaT040 as outlined above, followed by purging using Gubbins (v.2.1) (71) to obtain 3,774 core SNPs. Using BEAST (v.1.8.4) (74), the date of emergence of the *H*22 lineage and its distinctive ColV-positive sublineages was estimated. Briefly, the data were analyzed using the GTR and HKY substitution models with gamma-distributed among-site rate variation, with four rate categories. Both strict and relaxed molecular clock types were evaluated, as were the following coalescent tree priors: constant size, exponential growth, and Bayesian Skyline. The marginal likelihood for each model was estimated by using Tracer (v.1.5) (http://tree.bio.ed.ac.uk/software/tracer/) with Bayes factors. For each analysis, two independent runs of 500 million steps were performed, with sampling every 50,000th generation. The first 10% of each chain was discarded as burn-in. The Markov chain Monte Carlo samples were summarized using the maximum clade credibility topology by using TreeAnnotator v.1.8.4 from the BEAST package.

10.1128/mBio.00470-18.4 DATA SET S1  Metadata for genomes included from Enterobase. The data set includes information on strain ID, strain name, source, country of origin, continent, year of collection, SRA accession number, and ColV plasmid content. Download DATA SET S1, XLSX file, 0.6 MB. Copyright © 2018 Liu et al. 2018 Liu et al. This content is distributed under the terms of the Creative Commons Attribution 4.0 International license.

10.1128/mBio.00470-18.5 DATA SET S2  Metadata for Flagstaff isolates included in the study. The data set includes information on strain ID, sequence ID, isolate source, plasmid profile, resistance gene profile, ColV plasmid content, phylogenetic sublineage, meat type, *fimH* allele, *H*30 subclone, *gyrA* and *parC* allele, CTX-M content, and phenotypic antimicrobial susceptibility profile. Download DATA SET S2, XLSX file, 0.02 MB. Copyright © 2018 Liu et al. 2018 Liu et al. This content is distributed under the terms of the Creative Commons Attribution 4.0 International license.

### Phylogenetic placement of colistin-resistant E. coli ST131-*H*22.

Two ST131-*H*22 isolates from the Danish national surveillance program at Statens Serum Institut were identified retrospectively as carrying mobile colistin resistance determinants (*mcr-1* and *mcr-3*) (40, 41). Using the above-described methods, these isolates were screened for ColV plasmids and were analyzed for their phylogenetic placement within the global ST131-*H*22 collection.

### Statistical analysis.

Associations between ColV plasmids and antibiotic resistance phenotypes were assessed based on odds ratios (ORs) and a two-tailed Fisher’s exact test. Patient age was compared using a two-tailed *t* test with unequal variance. Patient sex and the prevalence of each UTI diagnosis were compared across *fimH* types by using a two-tailed Fisher’s exact test. A *P* value of <0.05 was considered statistically significant. All statistical analyses were performed in R (v.2.14.2) (75).
