# Supplementary figures and images for: Escherichia coli ST131-H22 as a Foodborne Uropathogen
Source: mBio. 2018 Aug 28;9(4):e00470-18. doi: 10.1128/mBio.00470-18 (PMC6113624; doi:10.1128/mBio.00470-18)

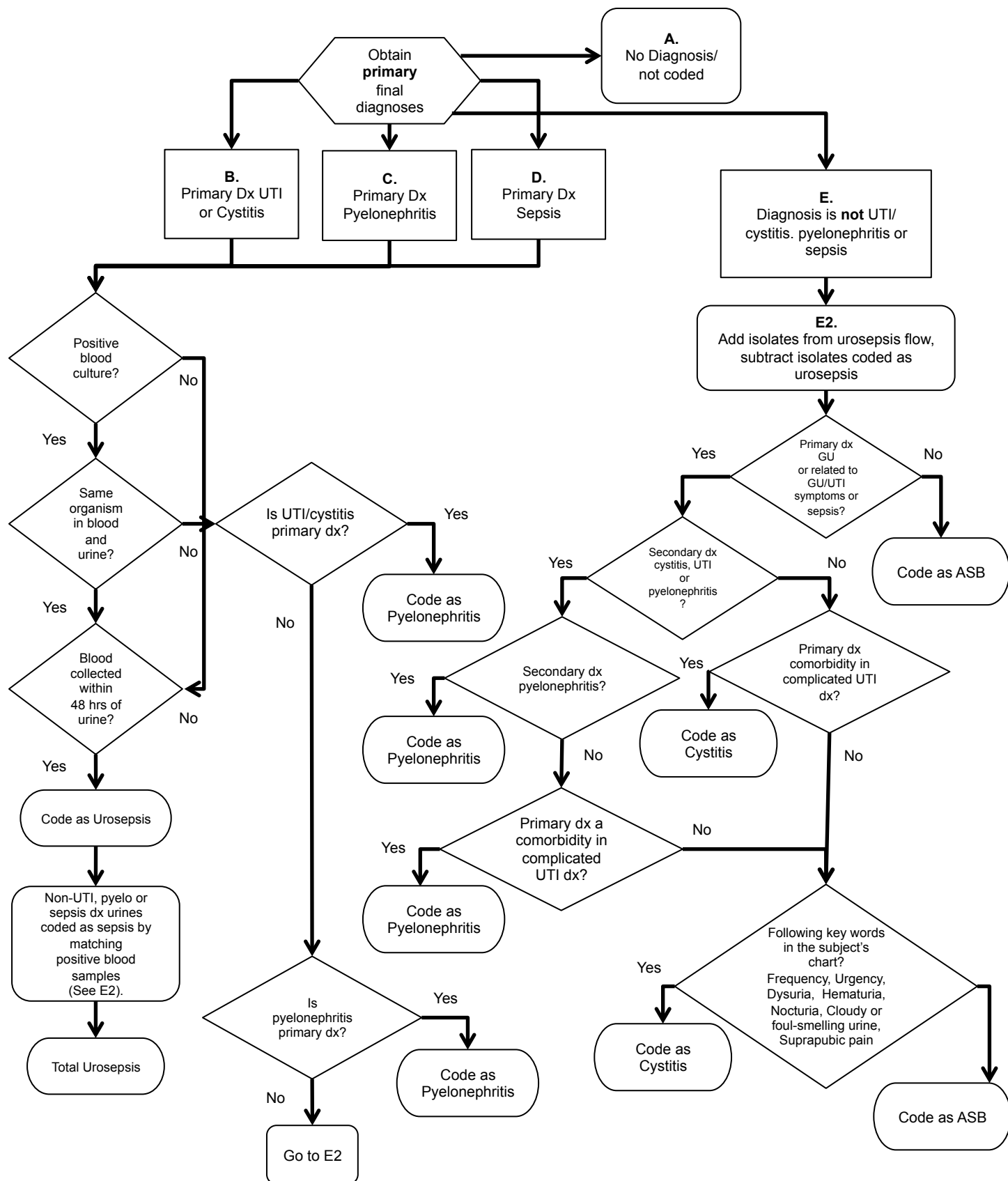

Supplement: FIG S1 [file mbo004184016sf1.pdf]

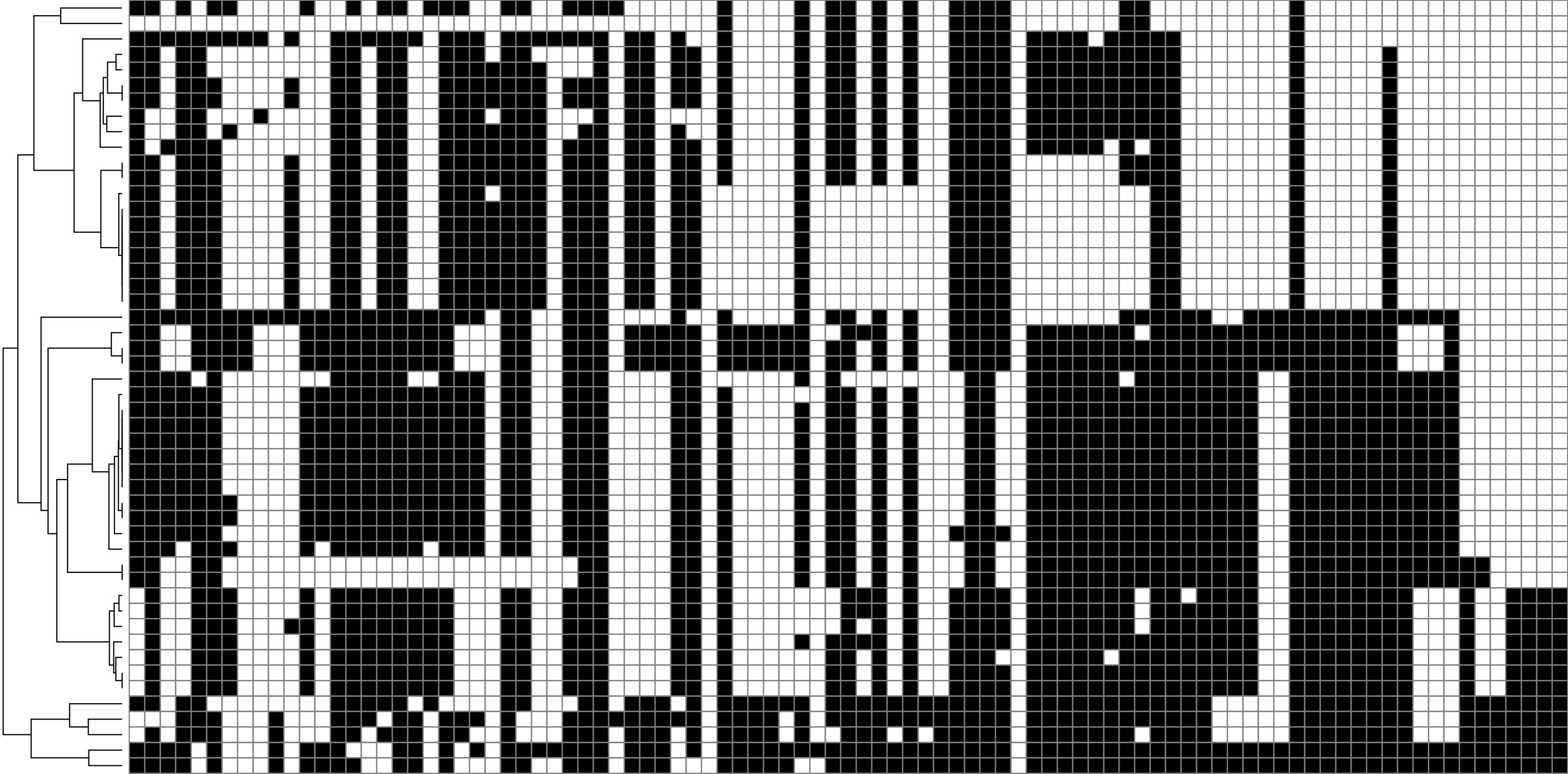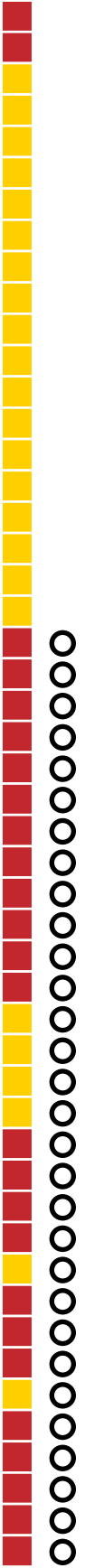

Supplement: FIG S2 [file mbo004184016sf2.pdf]

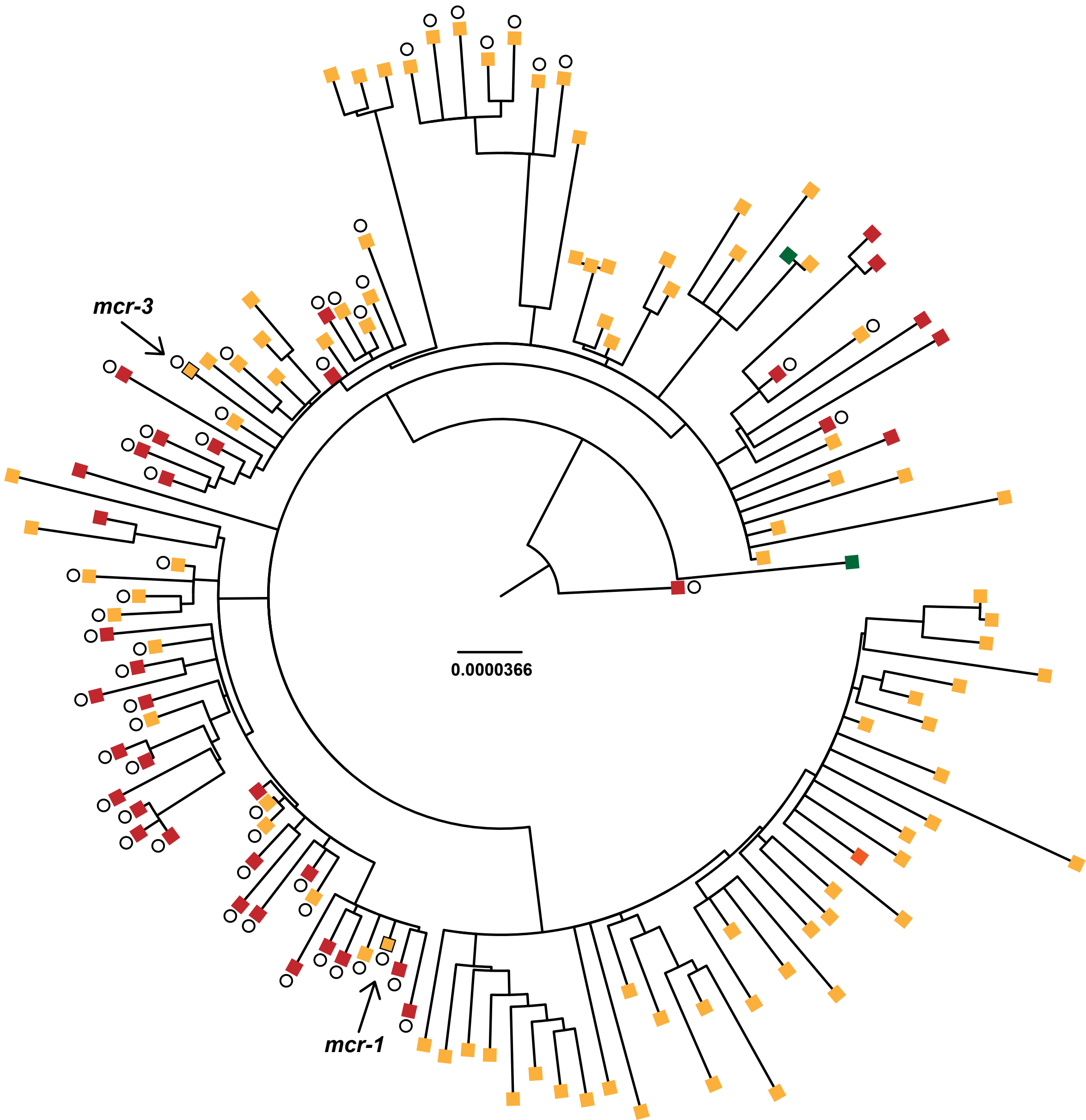

Supplement: FIG S3 [file mbo004184016sf3.pdf]
